# Are men clients, catalysts or controllers of family planning?

**DOI:** 10.3389/fgwh.2026.1590911

**Published:** 2026-02-24

**Authors:** Nour Horanieh, Alice Witt, Eloisa Montt-Maray, Marieme Fall, Elizabeth Larson, Thaïs Gonzalez-Capella, Beniamino Cislaghi

**Affiliations:** 1 Department of Global Health and Development, Faculty of Public Health and Policy, London School of Hygiene and Tropical Medicine, London, United Kingdom; 2 Department of Family and Community Medicine, College of Medicine, King Saud University, Riyadh, Saudi Arabia; 3 Center on Gender Equity and Health, University of California San Diego, San Diego, CA, United States; 4 School of Social Sciences, Makerere University, Kampala, Uganda; 5 Institute for Development Studies, Brighton, United Kingdom

**Keywords:** contraception, family planning, male engagement, men, reproductive health

## Abstract

**Background:**

Family planning (FP) has moved from being used as a tool for population control to promoting individuals’ and couples’ reproductive rights. The narrative surrounding FP focuses on how providing modern contraception empowers women to control their fertility and space their children. However, the role of men in the decision-making process of women's access, and use of FP services is under-examined. Although international health organisations insist that FP programmes ensure free choice for both men and women, most programmes primarily target women. This paper explores the role of men in FP programmes and services in the Global South.

**Methods:**

We conducted a qualitative study with 31 professionals from the international FP community, including academics, NGO workers, FP advocates, government officials, and international funding representatives. Interviews were conducted virtually and transcribed verbatim. Thematic analysis was applied to the dataset.

**Results:**

Participants did not have clear or consistent opinions about the role of men in FP service provision. Yet, all emphasised the need to prioritise women's reproductive health needs within FP programmes. Almost all reflected on men's role in the decision-making process around FP, emphasising how power dynamics between men and women in heterosexual relationships can limit women's autonomy. Some argued that men should have no role in FP programmes, as they were described as “controllers of” women's reproductive choices. Some participants reflected described men as “catalysts” and encouraged their engagement in programmes to support women in accessing, using, and continuing FP services. Others argued that men should be viewed as “clients” requiring FP services themselves. While different roles for men were discussed, no consensus emerged on a single approach. Participants also described tensions between women-centered policies at higher levels and the social norms impacting care at the community level.

**Conclusion:**

International organizations recommend targeting both men and women in FP programmes, but most programs continue to focus on women. These findings explore the roles men play in FP decision-making and identify barriers to engaging men in FP programmes. They highlight the need for culturally sensitive, gender transformative approaches to address gender norms and ensure reproductive rights for all.

## 1 Introduction

Family planning (FP) is an ancient aspect of human societies, deeply influenced by evolving cultural, religious, and political contexts. Reproductive decisions are often interwoven with moral and ethical considerations, making reproductive choices complex and deeply contextual. Historically, family planning was a personal or socio-familial responsibility, with ancient practices including fertility rituals, contraception methods for both men and women. Religious views on FP methods vary by method and gender and further reflect the influences of moral, ethical, and contextual factors, as well as social norms, on the reproductive decision-making process for both men and women (1, 2).

As societies modernised, state-funded FP programmes, influenced by neo-Malthusian beliefs, were implemented to curb the growing population (3). Many of these programmes faced criticism for employing coercive practices that disproportionately targeted communities in the Global South. Feminist academics and human rights' activists often highlighted the ethical violations and inequalities in these settings (4). Feminist movements have played a pivotal role in advocating women's reproductive rights and in prioritising women as the main beneficiaries of SRH services and FP programmes. The feminist movement went through several waves, from first-wave focus on access to abortion to second-wave emphasis on birth control (5). However, these movements, often Western-centered, drew criticism for sidelining women from non-Western cultures where larger families may be necessary socially or economically. Third and fourth-wave movements embraced intersectionality more openly to create more inclusive frameworks and promote reproductive justice for all individuals (6, 7). Nonetheless, critiques persist that global feminist agendas overlook the needs of women in crisis zones or underserved regions and may still lack intersectional perspectives when designing and implementing sexual and reproductive health (SRH) interventions including FP services (8). Furthermore, Western feminist scholars have been critiqued as looking at women in the Global South as a homogenous “other”, often described as constrained by family and tradition, as well as domesticated, uneducated and victimized and hence requiring liberation (9). Additionally, African feminisms usually frame the role of men and women as complementary that priorities community well-being and collaborative gender relations rather than competing roles (10, 11). They also address gender issues within broader systems of oppression, such as colonialism, racism, and political corruption, moving beyond the narrower focus of Western feminist frameworks (12, 13).

Today, the majority of FP programmes almost always target women exclusively and measure success in relation to contraceptive uptake among women (14). However, international organisations recommend targeting men and boys in addition to women and girls in FP programmes (15, 16). This discrepancy highlights how reproduction, as well as contraception, is often positioned as a female responsibility, even though reproductive decision-making often involves both partners (17, 18). Global development goals underscore the need for comprehensive FP services that “leave no one behind” (19), yet existing indicators largely measure women's contraceptive use within committed relationships, neglecting the role and concern of male partners in these settings, as well as single men and women.

In Francophone West Africa (FWA), family planning has historically faced unique sociopolitical and cultural challenges. These include entrenched patriarchal norms, religious interpretations, and limited access to reproductive health services (20). Despite these barriers, the Ouagadougou Partnership, launched in 2011, marked a turning point for FP efforts in the region. It brought together nine Francophone countries: Benin, Burkina Faso, Côte d'Ivoire, Guinea, Mali, Mauritania, Niger, Senegal, and Togo, to strengthen national FP strategies (21). By 2015, the partnership exceeded its initial goal of reaching one million additional contraceptive users. Building on this momentum, a new target was set to reach 2.2 million more users by 2020. While these achievements reflect political will and growing commitment, ongoing challenges include limited male engagement, weak health systems, and a shortage of context-specific evidence (21). These complexities highlight the need for a deeper understanding of how reproductive decision-making processes and policies can equitably address the roles and responsibilities of both men and women in whatever relationship they may be in.

There are many challenges in determining who should be and is responsible for reproductive and contraceptive decision-making, particularly as gender norms can dictate and determine who holds power over these matters. Social and gender norms often position women as the primary stakeholder in reproductive decision-making since they carry the biological roles of pregnancy, childbirth and breastfeeding. These roles combined with the disruptions in women's employment, education and financial independence, further highlight the need for women's reproductive and contraceptive autonomy. However, the responsibility of reproductive decision-making and childrearing goes beyond that of the biological roles of women. In many settings, men may bear the financial burden for supporting a family and both parents having emotional, social and familial roles in their children's lives. Additionally, men have their own reproductive health needs and desires which may include and are not limited to infertility treatments and family planning methods. A reproductive justice framework challenges the female-centred model of FP service provision, advocating for a shift beyond a purely rights-based approach. As it includes the protection of everyone's reproductive rights that includes legal rights to FP services and their rights to choose if and when to have children and with which partner, it also emphasises a holistic and intersectional approach (22–24). This framework situates reproductive rights within a broader social framework (25), ensuring that the global drive for reproductive health aligns with, rather than clashes with local cultural values, by addressing and working with them. Addressing men's roles in family planning alongside women's is essential for fostering a more inclusive approach that acknowledges the shared responsibilities and diverse needs of both partners.

Involving men in FP programming through community-based interventions and educational campaigns, has been reported to enhance women's access to services as well as foster mutual reproductive decision-making (16, 26, 27). Despite this evidence, most FP programmes remain predominantly women-centered, reflecting a tendency to overlook men's roles as partners or independent stakeholders in reproductive health and FP programmes. While the inclusion of men in FP programmes is often framed as a means of improving outcomes for women, this approach may reinforce gendered assumptions that may miminise men's reproductive health needs or position them solely as barriers to women's autonomy. Viewing men either as obstacles in FP or as default decision-makers risks oversimplifying the nuanced dynamics of reproductive health decision-making, which are shaped by complex intersections of cultural, social, and relational factors. Hence, this paper aims to explore the views of stakeholders from the international family planning on the role of men and their inclusion in family planning as well as exploring how their involvement can contribute to more effective and equitable FP programme delivery. By investigating the opportunities and barriers to integrating men into FP programmes, we seek offer insights into how FP initiatives can better meet the needs of both men and women.

## 2 Methods

### 2.1 Study design and setting

This paper is part of a larger, multi-level project examining ethical issues within the international FP community. The study presented in this paper represents the global level of this project, focusing on the perspectives of international stakeholders within the FP community on ethical challenges of FP programmes in FWA. We also conducted additional studies at regional and local levels, which captured the views of FP programme implementors in FWA, as well as community members, i.e., men and women living in Senegal on their views of FP programmes in their country. Peer-reviewed papers from the latter two layers will be published separately.

The main objectives of the global level study were to explore:

The relationship between sexual and reproductive health targets and women empowerment.
Funding challenges faced within FP programmes; women's representation in design and implementation of FP interventions.
Gender norms affecting women's access and use of FP services.
Ethical challenges faced by those working in the field and potential solutions.

During our interviews, questions related women's representation and gender norms in FP programmes uncovered different views on the role of men within FP programmes. Hence, this paper presents participants' perspectives on the role of men within FP programmes.

We employed a qualitative methodology using 31 semi-structured interviews. We conducted the interviews virtually between October 2020 and January 2021. The interviews lasted approximately 45 min and were audio-recorded with the permission of the interviewees. Interviews were conducted in either English or French depending on the interviewee's preference.

### 2.2 Sampling

We utilised a purposive sampling strategy to identify stakeholders from the international family planning community. We interviewed 31 stakeholders representing five categories: academics (9), advocates (5), government officials (4), NGO personnel (5) and funding agencies' staff (8). Participants were geographically distributed across North America (10), Europe (11), and Sub-Saharan Africa (10), of which 9 were in Francophone West Africa. Details of the participants are available in Table 1. Key informants, such as academics, funding agency workers and advocates, were instrumental in helping the research team in identifying and recruiting participants. Using a snowball sampling strategy (55) we recruited participants until we reached saturation. The research team contacted participants directly by email to request their participation. Consent forms and information sheets were shared in advance of each interview and all interviewees gave written consent to participate. Although the broader project is situated in FWA and includes participants from both the Global North and Global South, the sample for this study is weighted toward actors based in the Global North. This reflects a purposeful design choice since our analysis focuses on sites of decision-making power in FP programme funding and design that remain disproportionately located in the Western countries. Complementary analyses of other stakeholders from the region will be available in forthcoming papers that centre the perspectives from FWA to address this imbalance.

**Table 1:** Participants’ characteristics.

Number	Gender	Profession	Geographic Location
P 1	Female	Advocate	North America
P 2	Female	Funder	North America
P 3	Female	Funder	West Africa
P 4	Female	NGO	Europe
P 5	Female	Funder	Europe
P 6	Female	Funder	Global
P 7	Male	NGO	West Africa
P 8	Male	Academic	West Africa
P 9	Female	NGO	Sub-Saharan Africa
P 10	Female	Advocate	Europe
P 11	Male	Advocate	Europe
P 12	Female	Academic	Europe
P 13	Female	Academic	West Africa
P 14	Female	Advocate	North America
P 15	Female	NGO	Europe
P 16	Female	Academic	Europe
P 17	Female	Academic	Europe
P 18	Female	Government official	West Africa
P 19	Female	Academic	North America
P 20	Female	Academic	North America
P 21	Female	Academic	North America
P 22	Male	Academic	Europe
P 23	Female	NGO	West Africa
P 24	Female	Government official	West Africa
P 25	Female	Government official	West Africa
P 26	Female	Government official	West Africa
P 27	Female	Funder	North America
P 28	Female	Funder	North America
P 29	Female	Advocate	North America
P 30	Female	Funder	Europe
P 31	Female	Funder	Europe

### 2.3 Data collection methods

We developed the topic guide based on a scoping literature review aimed at understanding how FP interventions in sub-Saharan Africa address the ethical challenges identified in the literature (14), as well as consultations with the key informants. Next, we sought feedback from key informants and adjusted the guide according to their feedback. We piloted the topic guide with two members of the family planning community, to ensure the flow, clarity and relevance of our questions and themes. Our topic guide included open ended questions and related to the ethical challenges of FP programmes and potential solutions to them.

### 2.4 Data analysis

BC, NH, EM and AW conducted the interviews in the preferred language of the interviewee, either English or French. Each interview was attended by at least two members of the team to ensure all questions were asked and proper probing was conducted. All interviews were anonymised and transcribed verbatim in the original language, either English or French. French transcripts were translated by French-speaking members of the research team into English to allow all team members to take part in the analysis process. French-speaking members of the team, AW, MF, and EL, revised all the translated transcripts to ensure accuracy and preserve contextual meaning. Data analysis followed a thematic analysis approach and used NVivo software to code the transcripts thematically. The coding process was conducted in multiple stages. Initially, an inductive approach was adopted. EM, AW, MF, AZ-A and NH openly coded a subset of three transcripts to identify key words and emerging themes. Once data saturation was reached, the team developed a codebook, which was used for a second round of coding of a further twelve transcripts. To ensure consistency, regular meetings were held between the team members to cross-verify the codes and guarantee an agreement on the meaning of the codes. The codebook was then refined by AW and used to all the transcripts. Each transcript was double coded by two team members. Regular analytic discussions were held throughout the coding process to compare interpretations, resolve discrepancies, and refine themes. Intercoder reliability and consistency was ensured through iterative dialogue, and reflexive engagement. EM, AW, MF, AZ, NH, EL and BC analysed the data. NH wrote the manuscript. All authors reviewed the final manuscript and provided detailed feedback. Yet, potential biases were considered, mainly due to snowball sampling which may have resulted in overrepresentation of participants within interconnected professional networks. Furthermore, despite careful review, translations from one language to the other may have introduced subtle shifts in meaning. The team's diversity across geographic regions, cultural backgrounds, and professional experiences helped mitigate interpretive bias and enabled critical engagement and reflexive discussions throughout the analysis process.

## 3 Ethical considerations

All participants were anonymised, and interviews were assigned unique identification codes. Personal information of participants, as well as their organisations were omitted to prevent deductive disclosure. Participants were informed that collected data may be used in published academic papers and reports and we ensured that no identifying information would be published. All data is managed in accordance with the Data Protection Act 1998, and the LSHTM Information Security Policy and Research Data Management Policy. Only members of the research team have access to the anonymised data that is saved on a secured server. Ethical approval was provided on September 1st, 2020, by LSHTM research ethics committee, reference number: 22553/RR/21111.

## 4 Results

Throughout our interviews we asked participants their views on the role of men in FP programmes, how they perceive the effect of gender norms on women's fertility desires, and if and how they reflect on these roles and norms in their work. Although sometimes the conversation organically led to discussing the role of men in FP programmes, several interviewees seemed to be caught off guard when asked “what about men?” and changed their stance multiple times throughout the discussion. Many of our interviewees considered their own personal lives and reproductive decisions within their own intimate relationships, as well as their professional experience on the ground.

Honouring and protecting women's fertility desires seemed to take the top priority within all of our interviews. Even so, almost all participants acknowledged the complexities of reproductive decision-making, highlighting how they are shaped by social and gender norms as well as individual desires, interpersonal and community-wide relationships and cultural and legislative factors. Men's reproductive desires and role as potential FP service users were, in most interviews, described as secondary to women's, but not always.

Various roles for men in relation to FP programmes were outlined. In some cases, men were described as meriting no role in family planning decision-making, which was perceived as a women's-only domain or something which must be protected from male control. In other cases, participants described the role of men as “catalysts”, individuals to engage in FP programmes to promote women's access and adherence to contraception. In a few instances, participants stated that men should be viewed as FP programme clients, whose own reproductive desires and needs programmes should aim to meet.

We first demonstrate the global FP community's views on the complexity of reproductive decision-making and obstacles to FP programmes engaging men. We will then present the participants' views of men's roles as controlling entities, clients, and catalysts or collaborators within FP programmes.

### 4.1 Reproductive decision-making

Participants described the complexities surrounding fertility desires and the power dynamics within reproductive decision-making among couples especially when there is a discrepancy between what partners want in relation to expanding their family or limiting it. Many participants warned against framing the reproductive power dynamic as a battle of the sexes. Rather, they suggested encouraging a shared decision-making process that can foster collective empowerment for both men and women.

I've always had a problem with the words some of the women I know have told me “We have to take the power from men”. And I said “That doesn't work. That is not the way to word this. It's not a battle. It has to be that men realise that by giving some of this power they're all empowered more”. And that's the thing. That the sum of this negotiation is improved empowerment for both. And that's the key… It's not a zero-sum game. P27, Female funding Agency Worker, North America.

One participant emphasised that decision-making has to go both ways, telling us that she asks people who say that “The woman has to be the absolute one to decide” this question:

“Well, if a man wants to get a vasectomy, does he have to get his wife's or partners permission?”, and people are taken aback a little bit like “Oh, okay, well, it has to go both ways”. P1 Female Advocate, North America.

However, this participant clarified that her reflection was specific to decision-making within heterosexual, committed relationships. Other types of relationships would have different barriers and complexities related to social norms, religious views or legislative regulations. One participant provided us with an example of barriers single women who want to access FP services may face.

For younger women that are not married, they may not want to use the method openly or they may want to secretly have access to those methods. Some of them go to private health facilities or patient medicine vendors that are far away from them so that people around where they live will not know they are using a method. P13, Female academic, Sub-Saharan Africa.

Participants emphasised that the role of men in reproductive decision-making is made more complicated when there is an unequal power dynamic, which is often conditioned by social and gender norms that give more decision-making power to men. Hence, some underlined that empowering women to promote their family planning decision-making capacity must be a priority. A participant was reflecting on how she envisions the ideal FP programme being women-centred and treating women holistically and underlined that achieving this remains a challenge in the current programme funding environment.

It would be nice if we looked at this [FP] holistically, if we centered women's interests. I think where it gets tough is that a lot of the funding comes from more conservative sources who don't care as much about women's empowerment. P29, Female FP advocate, North America.

Other participants highlighted the financial role that men play in traditional marital relationships in specific contexts, that limits women's purchasing power and possibly access to family planning services.

Well, in our country, when you are married, it is the man who is responsible for the family, he has the decision-making power, he has the power to decide the size of his family. So the wife just submits. It is the man who has the purchasing power, so it is when the man wants it that the woman uses health services. P25, Female government worker, West Africa.

Participants highlighted that in some contexts, where men have the ultimate decision-making power, some women use contraceptive methods covertly, without their partner's knowledge and face fear and pressure of being forced to stop the contraception once the husband becomes aware of its use. However, they reflected that this situation was not considered ideal, and that involvement of men in joint decision-making would be preferable.

In most African countries the last word often goes to the husband…, if the wife does not consult the husband, once the husband is aware of this planning, often the wife is obliged to stop the planning because it is not the opinion of the couple. So the involvement of men here is very important for us. P26, Female government official, West Africa.

Other participants challenged FP programmes' default targeting of women only and identified how women-only FP centres end up excluding men from attending with their partners. A female NGO worker described an initiative that renamed maternity clinics to “family clinics” to allow both partners to attend and encourage men engagement in FP services. Following this change, healthcare providers found men started attending clinics with their partners and vocalised their fertility desires and openness to utilise contraception to reach their ideal family size.

What we saw is that of course men want to be involved and it's not that they want to have five, twenty children, they want to keep it at a manageable scope. P4, Female iNGO worker, Europe.

Other participants critiqued what they described as the feminist perspective that prioritises women's reproductive needs over men's.

I'm strongly against the view that's quite prevalent in some feminist circles that men have a hegemony, ultimate control over childbearing. I think it's much more nuanced than that. There probably are some patriarchal societies with big age differences between husband and wife where the man's desires dominate what happens. But I think in most societies it is roughly equal P22, Male Academic, Europe.

Similarly, a few participants highlighted how assumptions that family planning is women's-only issue have led FP programmes being inadequately set up to cater for men's reproductive needs and involvement. Participants further highlighted some of the logistical barriers to including men within FP programmes, which include the heavy focus on medicalised forms of female-targeted contraception and measures of programmes success being limited to a focus on uptake of these forms on contraception among women.

…you're coming from a rights/empowerment base and you really want to make sure women have, their own choice and knowledge, and that created a lot of focus on women. And then in terms of measuring [uptake of modern contraception], men were left behind and forgotten and that's now creating new problem. P31, Female Funding agency worker, Europe.

Participants frequently underlined that engagement of men in FP programmes was a complex, difficult and tricky issue, and suggested this as a reason why it is de-prioritised on the family planning agenda. However, many participants insisted that the active exclusion of men from FP programmes was “*one of the biggest mistakes we made as family planning people*”*.* P27, Female funding Agency Worker, North America.

Since engaging men in family planning programmes was described as a complex issue, participants outlined several roles that men could play in FP programming. In the next section we present the different ways participants described men's reproductive power and linked it to the suggested roles men should play within FP programmes: men as controllers of women's reproductive autonomy, men as catalysts to women's access and use of FP, and men as independent clients of FP services.

### 4.2 Men as controllers

Within many of the discussions with our participants, the control men have over women's reproductive choices and decision-making capacity was repeatedly highlighted as an issue and structural obstacle to women's access and FP autonomy in many contexts.

Men's control over our reproductive decisions and our ability to make those choices is a huge structural problem. P2 Female FP advocate, North America

Some participants provided examples of extreme male control over women's reproductive health to strengthen the argument for excluding men from FP services.

You've all heard the stories about men that cut the implants out of their [women's] arm and pull the IUDs out. How do we deal with this? P3, Female global funding agency worker.

The patriarchal control over women's reproductive autonomy was also described in relation to religious rulings, legislations, economics and social norms.

[In Senegal] Today we have a very strong patriarchal society. There is also the weight of religion, there is also the weight of morals and customs. These are all extremely important weights…There is also the economic problem, because these are extremely important issues. We cannot approach family planning from just one angle. We need several axes to talk about health issues today. A8 Male NGO worker, FWA.

In some settings, a pregnant woman is considered to be two lives, yeah? So, you know, in legal context if something happens to that person you have hurt two lives… For many people that creates tension. P6, Female Global funding agency worker.

One participant described how mothers-in-law embody the patriarchal norms of their social contexts by restricting women's reproductive autonomy, which forces many women to access FP services without the knowledge of the husband or larger family.

In Madagascar because the woman has no choice. It's the mother-in-law who decides on family planning, or even if she wants to do it, the family-in-law is opposed, the husband is opposed. And so, she does family planning in secret. P9, Female NGO worker, Sub-Saharan Africa.

Many participants linked women's reproductive autonomy to empowerment and freedom and hence strongly argued against male involvement in FP programmes. One participant stated:

I hate to talk about male engagement because I do feel like women should be independent and autonomous and free… I want every woman to be able to make that choice and to control her fertility, because I feel like controlling her fertility is part of being empowered and claiming your rights in this world. P3, Female global funding Agency Worker.

Others highlighted that decisions around whether men should be involved should be left to the woman themselves, especially since they carry the main burden of childbearing.

It would depend on the woman; it should be really a differential approach. Where, if this woman wants to come in for the service without the husband knowing you shouldn't try to convince her to bring the husband. If the woman wants to come with the husband, fine. Because the husband is supportive. P11, Male FP advocate, Europe.

Furthermore, one participant highlighted how FP services should not only cater to women in committed heterosexual relationships, hence women's desires in relation to men's involvement should be respected and prioritised when designing FP programmes.

That's [the role of men is] something that's been debated in the family planning community over and over again and different theoretical frameworks have been developed for it…couples [or] the family unit and women's relationships should be looked at holistically, but women obviously are most impacted by reproduction, by childbirth. Women generally bear the brunt of raising kids and the economic and health impacts. Also, women have multiple partners sometimes, it's not just a heterosexual monogamous family unit. So, I wish that the general accepted frame was “Ask women what they, how they want their husbands to be involved, if at all” and then design your programme according to what they want. P29, Female FP Advocate, North America.

Similarly, participants argued that if a woman's choice is to use covert contraception, the FP programme should be equipped to fulfil that choice.

You should think about her own right as a human being and her health. So, the service providers should consider that if the woman wants to take the FP method without the husband knowing, they should provide a service to her. But then they should really follow what women decide. P11, Male FP Advocate, Europe.

### 4.3 Men as catalysts

Many of our participants highlighted men's significant reproductive decision-making power within most settings and rather than focusing on diminishing their power, using it to women's advantage was the suggested way to go.

Expecting women to be empowered in all settings and demand that they're going to make decisions is a little bit unrealistic and culturally naive. In a lot of places where men are the decision makers, for better or worse, it's not that we're condoning that, but you have to start where you are in the culture and build up for more autonomy. P1, Female FP Advocate, North America.

Interviewees highlighted the tension between the ideal of empowering women by protecting their reproductive autonomy and the reality of achieving this in the context of complex cultural, social and legislative factors. Thus, participants underlined the need for programmes to understand and be grounded in addressing culture-specific nuances regarding FP decision-making, as well as gender and social norms.

We don't all live in the same society, and we don't live all in the same way. So what works best in that community? And when you go into a community and study it, are the men a detriment? Can they be won over? P3, Female Funding Agency worker

Those working in West Africa were more reflective about local norms, highlighting how in practice, men have ultimate decision-making power about fertility. As a result, many of those working in that region insisted that male engagement by FP programmes was necessary to ensure women can safely access and continue using FP services. They presented men's role as that of “catalysts”, who once they are supportive, will make women more likely to use methods or support them in their decisions to access FP services.

Men in Nigeria have control over the sexual life of women. So, if you mobilise the man, you have already mobilised most women in Nigeria. So, if family planning continue to target women, many of those interventions may not work- A9 Male Academic, Sub-Saharan Africa.

In most African countries the last word often goes to the husband. When the couple is involved, that is to say the husband is involved in the decision-making regarding family planning, and that the husband adheres to the planning, it goes the longest. So often in the couple, this is an observation, if the wife does not consult the husband, once the husband is aware of this planning, often the wife is obliged to stop g because it is not the opinion of the couple. So, the involvement of men here is very important for us. P26, Female government official, West Africa.

Participants highlighted the role of behaviour change interventions to address social and gender norms and increase men's support of FP services.

When we're talking about men as partners, I think it's hugely important to address those social norms, if you want a healthy family and a wealthy family then everybody goes to school and finishes their education before they have kids. And you give space between births to improve the health of your wife whom you love and your children. That's where I think the male partnership really comes into play, working on gender norms. P15, Female NGO worker, Europe.

A government official from Guinea described how their country was adopting successful programmes from neighbouring countries that target men to promote family planning use and encourage couple decision-making to space children within marriages.

In other countries like Niger, they create spaces for informing and raising awareness among men, which they call the “husband schools” to inform and raise awareness of the advantages of FP, and get them to be keen on the provision of these services for women. And for themselves, because we can get them to use condoms to prevent pregnancies or sexually transmitted infections, and for the couple. The man must feel involved in the health programme. P25, Female government worker, West Africa.

The same participant also suggested targeting specific sub-groups of men to ensure women's family planning use is protected and supported.

There should be objectives for certain target groups, which are young people- men. I'm going to talk here about the husbands who are very reluctant—and there are also religious leaders. So, objectives targeted towards these groups would be very interesting to look at that so that provision of FP services would be effective, there would no longer be any reluctance P25, Female government worker, West Africa.

### 4.4 Men as clients

Some participants underlined that men themselves should be targeted by FP programmes as clients, either as contraceptive users themselves or as joint decision-makers with their partners. They emphasised that women often want their partners to be part of the decision-making process.

We know that when couples communicate about childbearing and contraceptive use and partners are supportive then, they're more likely to use contraception… And many women want their partner to be involved. P2, Female funding agency worker, North America.

In many of our interviews, participants expressed a frustration with men's absence from the FP decision-making process. One participant highlighted the responsibilities and stakes men have in child rearing, and therefore the need for them to be involved in reproductive decisions. Furthermore, the exclusion of men was sometimes perceived as a Western feminist stance that insists that reproductive rights are a women's only issue.

What men want, the number, the timing of children, is as important as that of women. Because men bear the main responsibility for bringing in enough money to support that extra child… I think much of the rhetoric from the West that I see at conferences where what men want and men's aspirations reproductively is completely sidelined and it's portrayed entirely as a woman's issue, is fundamentally wrong and counterproductive in societies where men's voices are very strong. P22, Male Academic Europe.

Similarly, men were also described as needing to be treated as individual clients since they are targeted through marketing strategies for contraception. Although many of the modern contraception products are designed for women, men were described as having a social and financial stake in taking care of their partners' and families' wellbeing was described as reason to target FP demand generation activities at men too.

Men respond to demand issues too. Even though the product is not for them, they do purchase. If you look at marketing experience and research, they do respond to products that are not for them, for personal use, but benefits of a family. P21, Female Academic North America.

The way to involve men was described as not just simply including them as companions to their wives during clinic visits for shared counselling sessions, but as equal recipients of tailored education counselling whose aim is fostering joint decision-making between partners.

I believe that FP programmes could do more to involve men. It doesn't mean that the husband needs to be in the counselling consultation with the women to make the decision. It means the husband should have access to the same level of information about the methods, about the benefits, and the counselling is not just who decides which method, but how they talk to each other about joint decisions. P21, Female academic, North America.

One participant highlighted the need to recognise men as autonomous, independent clients of FP services, rather than extensions of the couple, while encouraging meaningful conversation between partners. At the same time, she stressed the need to ensure women retain full access to the services they desire without men's interference.

I don't like when men are seen as an appendage as opposed to a full human… There's a tendency to talk about male involvement in a way that was really demeaning to men. I don't think that's helpful… I think there's an important role for interventions that support couples to speak, but I wouldn't want to say that that authorisation in any way needs to be part of the access that women would have. P19, Female academic, North America.

Participants also highlighted that men can be contraceptive users themselves, for example through condom use. However, male-controlled FP methods are often under-discussed and under-promoted. A funding agency worker emphasised that social and gender norms change were needed to encourage men to take greater responsibility for FP.

I've had this argument over and over, saying if a condom is good enough to prevent HIV, how is it not good enough to prevent pregnancy? It's a matter of behaviour change… It's all about norms, fundamentally. P27, Female funding agency worker, North America.

## 5 Discussion

Our paper aimed to understand the role of men within family planning programmes from the perspectives of international stakeholders and experts from the family planning community. Throughout our interviews participants reflected on the complexities in reproductive decision-making between partners as well as the reproductive power dynamic and responsibilities for men and women. They highlighted that while joint decision-making between partners is an optimal ideal, there are challenges to realising this in practice, and the role of men should be considered in a context-specific or even relationship-specific way. The role of men was framed in three ways: as controllers whose excess decision-making power should be mitigated; as catalysts who could be used to promote FP uptake among women; and as clients, who could be engaged by FP programmes either as users or as shared decision-makers alongside their partners. The “Men as Controllers” approach assumes men dominate women's reproductive choices, leading to the insistence on promoting medicalised forms of contraception that usually bypass male control, placing the full contraceptive responsibility on women. On the other hand, the “Men as Catalysts” approach views men as potential allies in reproductive decision-making that should be utilised and suggests social solutions to increase men's support to women's reproductive decisions. However, this approach presents a challenge; resistance to shifting power dynamics. The last approach, which is the “Men as Clients” approach recognises men's own reproductive health needs and promotes a shared responsibility through improved access to services. Similarly, it risks reinforcing male influence in decision-making. Table 2 provides a summary of the definitions of different categories as well as examples of interventions.

**Table 2:** Definitions of categories.

Categorisation	Definition	Example of Intervention
Controllers	Men dominate women's reproductive choices	Providing covert contraceptive options to women
Catalysts	Men are allies to women's reproductive health and choices	Increasing men's support to women's reproductive choices including to access, use or not use FP services
Clients	Men should be seen as having their own reproductive health ineeds	Promoting a shared responsibility through improved access to FP services for both men and women

For the most part, participants articulated that fulfilling women's reproductive choices should be prioritised by FP organisations above all other actors' desires. This argument was made based on the fact that women bear the primary physical burden of pregnancy and childbearing, as well as risks associated with loss to education and employment opportunities (28–30). Participants felt it was particularly important to safeguard women's reproductive autonomy in instances where power dynamics in a relationship were imbalanced and highlighted that gender norms are a key driver of these imbalances. The effects of power dynamics, gender norms and economic power on household decision-making have been widely reported (31–33).

However, findings from this study challenge the assumption, often made in FP programme design and implementation, that women alone should be responsible for contraceptive and reproductive decision-making, and that men's needs and priorities are secondary (17, 34). This assumption is clear in how most programmes target women, especially through female-specific contraception, and how programme success is primarily measured based on increased contraceptive uptake among women (14, 35). This study demonstrates that men may wish to be involved in reproductive decision-making as they have responsibilities, particularly financial responsibilities, during child rearing. Examples from Nigeria and Kenya reveal how engaging men in FP decision-making fosters better communication and shared responsibility in FP decisions (34, 36). Moreover, meaningfully joint decision-making between partners within households has been found to improve spousal support, communication in relation to use of modern contraception (36–38). Furthermore, recent evidence from a multi-country cross-sectional study has shown that a large percentage of men, reaching 76% in some contexts, are interested and willing to try novel forms of male-specific contraception (39). The study also highlights female partners' trust in men to use and adopt novel forms of male-specific contraception. Yet, the majority of funding for contraception research and development continues to be disproportionally aimed at female contraception (40).

International initiatives like the WHO's *Engaging Men and Boys in Changing Gender-Based Inequity in Health* highlight the value of involving men in reproductive health to improve outcomes for all family members (27). Programmes targeting men and boys are praised as leading to increased contraceptive uptake, continuation, and maternal health improvements (16, 26, 41). There is an increasing acknowledgment of the need to develop interventions that involve men and boys to “promote family planning behaviours” (42). This was echoed by our participants who consistently suggested engaging and educating men through interventions that promote joint decision-making as well as increasing FP access and use for women. However, the role of men as catalysts, or as facilitators of FP demand generation among women, has interesting ethical implications. In particular, it can be argued that to be meaningfully gender-transformative, norms change programmes should be working to address gendered power imbalances for their own sake, rather than for the sake of achieving high-level FP goals regarding contraceptive uptake.

Beyond their roles as partners, this study presents men as potential clients of FP services who would benefit from male-focused services, such as counselling for reproductive life plans, discussions of intimate partner violence, and primary care services (15, 26, 41). Addressing men's health holistically not only benefits them but also promotes healthier partnerships and family outcomes (43). Furthermore, our participants highlighted how the focus on women-only contraception methods and the underutilisation of male methods like condoms may be a barrier to engaging men within FP services. Other studies have reported similar findings (44). Some of these studies also explored the engagement of men in joint contraceptive methods like the standard days method and showcased how these approaches can facilitate male engagement while promoting gender equity in contraceptive decision-making (45). Expanding the use of methods that encourage shared responsibility may help address the gendered division often seen in FP service provision.

Another key theme that emerged from this study is the importance of involving men in FP decision-making in ways that respond to context-specific gender norms and culturally sensitive practices. This finding align with the wider literature that recommends FP programmes promote male engagement and behaviour change regarding reproductive decision-making in a culturally sensitive manner (44, 46). Additionally, our findings highlight the necessity of applying an intersectional lens to the feminist approach upheld by the FP community. Participants expressed diverse interpretations of feminism, with some critiquing the belief that FP is a women-only issue and the excludsion of men from FP services. This resonates with critiques in the literature, which argue for more inclusive feminist frameworks that acknowledge and address men's roles without reinforcing traditional gender hierarchies. Additionally, these arguments emphasise the importance of listening to users of FP services to fully understand their reproductive desires and needs (47–49). By adopting an intersectional lens, FP programmes can better address the effects of gender norms, culture, and power dynamics and tailor FP services to consider different identities and contexts (50). These findings resonate with recent feminist discourse emphasising a gender-transformative lens, which argues that involving men should not be framed as a zero-sum competition between women's and men's health, but rather as a way to acknowledge that men are functional agents in achieving health outcomes for women while also having their own health needs. This approach calls for developing a shared understanding and framing of gender justice in global health and showcasing expanded conceptual frameworks to critically examine the validity and application of male involvement in SRH. Such perspectives deepen the interpretation of our findings and underscore the importance of moving beyond simplistic inclusion to transformative engagement that addresses structural gender norms (16, 51–53).

While much of the discussion around reproductive decision-making focuses on partners and systemic factors, the role of the child was conspicuously absent within our discussions. Participants reflected on decisions leading up to a child's birth but not on how the child's presence affects the lives of mothers, fathers, and families. The literature similarly rarely looks at the value of children for men except in divorce studies (54). This highlights the need for FP programs to adopt a reproductive justice framework that takes a holistic view of reproduction, placing the needs of men, women and children at the centre, while fostering safe environments for individuals, couples and families to fulfill their reproductive desires. Although our participants highlighted the complexities of incorporating men's reproductive health needs within FP services, some provided examples of successful programmes that achieved this like the example provided on renaming maternity clinics to family clcinics to ensure an inclusive and welcoming space for men. Hence, we call on the FP community and international organisations to dedicate greater consideration to the role of men in FP programmes they run, and to how they can be engaged in meaningful and equitable ways to realise the call for men and boys' involvement in FP in practice. Lastly, as the Lancet Commission on gender and global health argue, interventions should move away from understanding gender and health, as well as gender justice, as a zero-sum game, where women and men compete for SRH services and interventions. Instead, framing men as SRH and FP clients can be pursued as a broader strategy to improve overall health, as women and men's wellbeing are interdependent (52).

The primary strength of the study is the diversity of participants, who represented a range of geographic locations and professional backgrounds. However, a key limitation was that the majority of our participants (21 out of 31) were from the Global North. While we included participants from the Global South, particularly from FWA where the larger study is situated, this paper focuses on presenting the perspectives of those stakeholders that strongly influence funding and design of global FP programmes, who continue to be based in Global North institutions. Hence, we do not suggest that our findings should be interpreted as universally representatives, but rather highlight a dominant discourse shaped by structural power. Greater representation from the Global South would have provided a more balanced perspective, as it could have also offered more insights into how men are perceived and targeted within FP programmes, as well as highlighted more barriers to implementation related to local social norms and contexts. Complementary analyses within the broader project, to be published separately, centre the Global South perspectives and aims present the views of stakeholders working within the region of FWA, specifically, programme implementors and users, will address this limitation.

## 6 Conclusion

Our results highlight the complexities of reproductive decision-making within couples, which are influenced by power dynamics, gender norms, and systemic factors. While women often bear the primary responsibility for contraception, men's roles within FP programmes remain under-addressed and narrowly defined. It is important to address the assumption that contraception and reproduction are the responsibility of women alone. To achieve this, efforts are needed to address both the default assumptions that guide FP programme design, as well as entrenched patriarchal norms, systemic inequities, and contextual barriers that constrain progress toward gender equity. Our analysis highlights that context- and situation-specific engagement of men should be the priority, but that engaging men in joint decision-making and as FP programme clients, in their own right, can foster improved communication, shared responsibilities, and better health outcomes for families as whole as well as individual members. Moving forward, it is critical to design interventions in culturally sensitive and gender transformative ways that ensure protecting women's autonomy through challenging norms that may prevent meaningful joint decision-making as well as addressing men's own reproductive needs and desires.

## Data Availability

The datasets presented in this article are not readily available because we cannot provide the full transcripts of the study as there is a possibility of deductive disclosure risk. We are committed to protect the identities of our participants and hence cannot provide the full transcripts. Requests to access the datasets should be directed to nour.horanieh@lshtm.ac.uk.
